# Chinese Clinical Named Entity Recognition with ALBERT and MHA Mechanism

**DOI:** 10.1155/2022/2056039

**Published:** 2022-05-23

**Authors:** Dongmei Li, Jiao Long, Jintao Qu, Xiaoping Zhang

**Affiliations:** ^1^School of Information Science and Technology, Beijing Forestry University, Beijing 100083, China; ^2^Engineering Research Center for Forestry-oriented Intelligent Information Processing, National Forestry and Grassland Administration, Beijing 100083, China; ^3^Institute of Information on Traditional Chinese Medicine, China Academy of Chinese Medical Sciences, Beijing 100053, China

## Abstract

Traditional clinical named entity recognition methods fail to balance the effectiveness of feature extraction of unstructured text and the complexity of neural network models. We propose a model based on ALBERT and a multihead attention (MHA) mechanism to solve this problem. Structurally, the model first obtains character-level word embeddings through the ALBERT pretraining language model, then inputs the word embeddings into the iterated dilated convolutional neural network model to quickly extract global semantic information, and decodes the predicted labels through conditional random fields to obtain the optimal label sequence. Also, we apply the MHA mechanism to capture intercharacter dependencies from multiple aspects. Furthermore, we use the RAdam optimizer to boost the convergence speed and improve the generalization ability of our model. Experimental results show that our model achieves an F1 score of 85.63% on the CCKS-2019 dataset—an increase of 4.36% compared to the baseline model.

## 1. Introduction

Clinical named entity recognition (CNER) is a fundamental and crucial task in medical natural language processing problems. Researchers aim to identify and extract the clinical entity mentioned in electronic medical records (EMRs) and classify them into predefined categories (e.g., disease, symptom, and treatment). Additionally, extracting named entities from semistructured or unstructured EMRs is helpful for further research, such as building clinical decision support systems and medical knowledge graphs.

Recent developments of deep learning (DL) have led to their overwhelming performances in the field of natural language processing. At the same time, researchers have adopted DL methods on biomedical tasks [1–4]. Compared with traditional rules and dictionary-based methods or machine learning (ML) methods [5–7], DL methods have the advantage of stronger generalization ability and less reliance on rule design or feature engineering. In particular, the bidirectional long short-term memory with conditional random field (BiLSTM-CRF) method [8, 9] has achieved significant results in CNER [10–12]. However, the word-level BiLSTM model cannot solve the problem of error propagation caused by the wrong entity boundary recognition, nor can it make full use of the parallelism of the graphics processing unit (GPU). Also, the entities in Chinese EMRs have a unique and rigorous language structure [13], which makes Chinese CNER more challenging.

To solve the above problems, Strubell et al. [14] proposed an iterated dilated convolutional neural network (IDCNN) model for named entity recognition, which simultaneously improved training speed and accuracy. Gao et al. [15] used an attention-based IDCNN-CRF model for the CNER task and demonstrated the effectiveness of combining word order features and local context. However, this approach does not effectively integrate the contextual semantic information of a sentence, nor does it accurately represent polysemous words. Li et al. [16] proposed the BERT-BiLSTM-CRF model, which incorporated dictionary features and radical features of Chinese characters to improve model performance. However, the model's stringent requirements for the quality of dictionary and storage space limit its performance in actual scenarios. Fang et al. [17] developed an end-to-end neural network based on a multi-head attention (MHA) mechanism and two hint mechanisms for the joint extraction model of entities and relations. The model outperformed the state-of-the-art methods of joint entity and relation extraction.

For the Chinese CNER task, we propose the ALBERT-IDCNN-MHA-CRF model. This paper's main contributions are as following:

1. We fine-tune the ALBERT pretraining model to enhance the semantic representation.
2. We use the IDCNN model to encode the global information of the entity and speed up the training process.
3. We use a multi-head attention mechanism to capture the context information.
4. We use the RAdam optimizer to boost the convergence speed and improve the model's generalization ability.
5. The evaluation results show that our model achieves good performance on the CCKS-2019 datasets.

## 2. Related Work

At present, the methods for the CNER task are divided into three categories: rule-based and dictionary-based methods, ML-based methods, and DL-based methods [18].

Rule-based and dictionary-based methods have been mainly used in the early CNER system and related applications. They rely only on existing dictionaries and manually constructed rules, which cause problems of long system development cycles and poor portability for complex and diverse entities in EMRs. In contrast to the above methods, the ML-based method has good versatility, which regards the CNER task as a sequence labeling problem and uses a large-scale corpus to label each position of the sentence. Classical ML methods such as the hidden Markov model, maximum entropy Markov model, support vector machine, and CRF are widely used in the CNER task. Nevertheless, constructing a large-scale labeled corpus in the early stage is costly, and the high dependence on manual feature engineering is time-consuming.

Recently, methods based on DL have been successfully applied to the CNER task. The BiLSTM-CRF method achieved the most advanced performance on many CNER datasets. However, the time series-based calculation in the LSTM model could not achieve efficient parallelism, and it is challenging to capture the long-term dependence between characters in the face of long sentences. For large-scale electronic medical record corpora, there have been problems with high model complexity and slow training speed. Therefore, researchers have attempted to use the CNN method to effectively capture contextual semantic information while taking full advantage of GPU parallelism to improve the model efficiency.

Unfortunately, the above DL-based methods failed to distinguish ambiguous characters or words. For example, the character “清” (clean) has completely different meanings in the two sentences of “患者神志清、精神可” (the patient is conscious and in good spirits) and “于我院行淋巴结清扫术” (lymph node dissection in our hospital), but they would be mapped to the same vector in static word embedding representation methods (such as Word2Vec). So, it could not consider the contextual semantic information of the sentence.

In recent years, many pretrained contextual word embedding models have been proposed, such as EMLo and OpenAI-GPT. However, the above two pretraining models cannot simultaneously obtain the semantic information of the EMRs in the front and back directions. Bidirectional encoder representations from transformers (BERTs) solve the above problems well. For the CNER task, we only need to set the downstream task interface and use the relevant data to fine-tune the model to obtain a more accurate embedded representation of each word in the EMRs. Cai [19] first enhanced the semantic representation of characters through BERT, further inputting the word embedding into BiGRU-CRF for training, and finally achieved better performance. Zhang et al. [20] pretrained BERT on the corpus of Chinese clinical text and used the embedding as input features of BiLSTM-CRF to solve the breast cancer CNER problem, and achieved an F1 score of 93.53%.

BERT had excellent performance in CNER, which mainly benefited from its “overparameterized” nature. Owing to its millions or even billions of parameters, its computational efficiency is low, which greatly hinders its application in actual CNER systems. Therefore, researchers have begun to study on compressing BERT's size with an acceptable tradeoff on performance to speed up the training progress. Sun et al. [21] outlined a “patient knowledge distillation” method by compressing the model into a lightweight shallow network. Fan et al. [22] proposed LayerDrop, a structured dropout method, to train the transformer model. Without fine-tuning, they sampled subnetwork from the original model through a pruning strategy to generate a high-quality small BERT model. Shen et al. [23] proposed a new group-by-group quantization scheme and compressed the model with Hessian-based mixed-precision quantization. The ALBERT model proposed by Lan et al. [24] applied two parameter-reduction techniques to reduce memory consumption and improve the training speed of BERT while using a self-supervised loss to improve the training effect.

## 3. Materials and Methods

**Figure 1:**
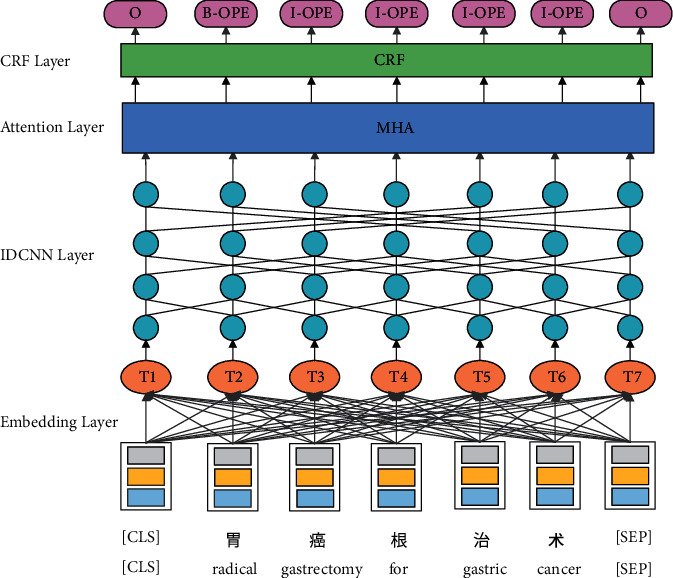
Main architecture of our ALBERT-IDCNN-MHA-CRF model.

Figure 1 illustrates the overall ALBERT-IDCNN-MHA-CRF network architecture of our model. First, for each Chinese character of EMRs, the character, sentence, and position features are computed by ALBERT. Second, we concatenate the three embeddings and feed them into the IDCNN network to extract the global features, and then input the embeddings to the MHA layer to capture the long-distance dependencies between characters by calculating the attention probability of sentences from multiple aspects. Finally, we concatenate the output vector of the MHA layer into a CRF layer, which constrains the dependency relationship between the prediction labels and obtains the best label sequence. To improve the generalization ability of the model, we add a dropout layer between the embedding layer and the IDCNN layer.

### 3.1. Embedding

Language modeling is a key concept in natural-language processing tasks. While BERT enjoys an outstanding performance in CNER, its overparameterization leads to a large memory footprint and time consuming.

Compared with BERT, ALBERT has mainly made improvements in three aspects: factorized embedding parameterization, cross-layer parameter sharing, and intersentence coherence loss, which remarkably reduces the total number of parameters and reduces the model's complexity.

**Figure 2:**
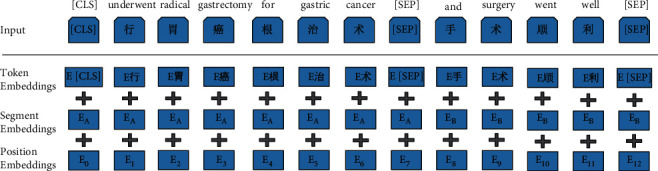
Input example.

For each word in the EMRs, the input representation of ALBERT consists of three parts: token embedding, segment embedding, and position embedding. Token embedding represents a word vector that can be either a word vector or a character vector in the Chinese language. Owing to the unique sublanguage characteristics and complex language structure of EMRs, we use character embedding for representation. Segment embedding is used to distinguish pairs of sentences. Position embedding is the position information obtained from the model learning. The calculation equation is:

(1) $$\begin{array}{c} PE_{\left(pos,2i\right)}=\sin\left(\frac{pos}{10000^{2i/d_{\text{model}}}}\right),\\ PE_{\left(pos,2i+1\right)}=\cos\left(\frac{pos}{10000^{2i/d_{\text{model}}}}\right), \end{array}$$

where *pos* is the position in EMR, *i* is the dimension, and *d*_model_ is the vector dimension after encoding.  Figure 2 shows an example of this input.

### 3.2. IDCNN

To effectively extract the text features of EMRs, while speeding up the training process and improving prediction efficiency, this study uses the IDCNN model for feature extraction. Dilated convolution was originally applied in the field of image processing. Unlike traditional CNNs, it uses the dilation width between the convolution kernels without the pooling operation to reduce information loss and increase the receptive field. The receptive field of dilated convolution is calculated as

(2) $$\begin{array}{c} F_{i+1}=\left(2^{i+2}-1\right)\times \left(2^{i+2}-1\right). \end{array}$$

Here, we use four identical blocks of dilated convolution. Each block has three dilated convolution layers with dilation widths of 1, 1, and 2. Thus, there are four iterations, where each iteration takes the previous result as the input. This parameter-sharing mechanism effectively prevents overfitting. As the number of layers increases, the receptive field increases exponentially, while the parameters increase linearly so that the receptive field quickly covers all input sequences. In the IDCNN model, the parameters of each layer are independent and of the same scale, which effectively reduces the parameters during training, and thereby speed up the training.

The IDCNN model encodes each character in EMRs and extracts the features in the text to generate corresponding feature vector. Although the encoded vector contains long-distance semantic features, these features share the same weight and cannot solve the problem of different correlations between characters. Hence, further feature extraction is required through the multi-head attention layer.

### 3.3. MHA

Since the entities in EMRs do not exist in isolation, there are specific dependencies between each other, accompanying a long interval between the characters of the entity. For example, in the sentence “患者因胃癌于2015-5-19于我院行胃癌根治术, 术后恢复良好” (the patient underwent radical gastrectomy for gastric cancer in our hospital on May 19, 2015, and recovered well after the operation.), “胃癌”(gastric cancer) belongs to the disease entity, and “胃癌根治术” (radical gastrectomy for gastric cancer) represents the operation entity. These two entities often appear in the same EMR, suggesting a certain dependence between them.

To capture this dependency, the model has to pay more attention to the characters dependent on the current character and assigns higher weights to these dependent characters and smaller weights to other irrelevant characters so as to recognize the entity type of the character better.

Here, we pick the MHA model for multiple self-attention calculations in order to learn relevant information in different representation subspaces. The MHA model also ensures parallel computing performance superior to recurrent neural networks. Scaled dot-product attention in the model is defined as

(3) $$\begin{array}{c} \text{Attention}\left(Q,K,V\right)=\text{soft}\max\left(\frac{QK^{T}}{\sqrt{d_{k}}}\right)V, \end{array}$$

where the query *Q*, key *K*, and value *V* are all in vector form, $1/\sqrt{d_{k}}$ is the *k*-dimension adjustment smoothing term, and the softmax function value is the normalization factor. We set *Q* = *K* = *V* when calculating self-attention, which represents the characters in the sentence.

In the CNER task, for an input sentence *X*=(*x*_1_, *x*_2_,…, *x*_*n*_), the output after IDCNN layer is *Y*=(*Y*_1_, *Y*_2_,…, *Y*_*n*_). For the output state *Y*_*t*_ of the *t*-th character in the sentence, the single-head self-attention calculation is performed using formula (4). A total of *h* calculations are performed, and the result of the *i*-th calculation is head_*i*_.

(4) $$\begin{array}{c} {\text{head}}_{i}=\text{Attention}\left(Y_{t}W_{i}^{Q},Y_{t}W_{i}^{K},Y_{t}W_{i}^{V}\right), \end{array}$$

where *W*_*i*_^*Q*^ ∈ *ℝ*^*d*_model_×*d*_*k*_^, *W*_*i*_^*K*^ ∈ *ℝ*^*d*_model_×*d*_*k*_^, and *W*_*i*_^*V*^ ∈ *ℝ*^*d*_model_×*d*_*k*_^ are weight matrices of the *i*-th calculation.

After concatenating the calculation results of these *h* times and performing a linear transformation, the result of the *t*-th character in the sentence is obtained, which is given by

(5) $$\begin{array}{c} MHA_{t}=\text{concat}\left({\text{head}}_{1},{\text{head}}_{2},\ldots ,{\text{head}}_{h}\right)W^{O}, \end{array}$$

where concat() is the splicing function and *W*^*O*^ ∈ *ℝ*^*hd*_*v*_×*d*_model_^ is the weight parameter.

### 3.4. CRF

The output of the MHA layer is the probability or score of each label corresponding to each character in the sentence. Denote the scoring matrix by *P*. If the label is modeled and output independently, the dependency between labels is ignored (for example, the “I-CHE” label cannot be immediately followed by the “B-DIS” label), which is essential information for the decoding module. Therefore, we introduce the CRF layer for label decoding, which constrains the dependency relationship between predicted labels to decode the global optimal label sequence.

For a given input sequence *X*=(*x*_1_, *x*_2_,…, *x*_*n*_), and the corresponding label sequence *y*=(*y*_1_, *y*_2_,…, *y*_*n*_), let *W* be the transition matrix, the evaluation score is defined as

(6) $$\begin{array}{c} S\left(X,y\right)=\sum_{i=1}^{n}P_{i,y_{i}}+\sum_{i=0}^{n}W_{y_{i},y_{i+1}}, \end{array}$$

where *P*_*i,j*_ is the score of the *i*-th character labeled as label *j*, and *W*_*i,j*_ is the state transition score from label *i* to label *j*.

Given *X*, the conditional probability of the sequence label *y* is calculated through the softmax function:

(7) $$\begin{array}{c} P\left(y|X\right)=\frac{e^{S\left(X,y\right)}}{\sum_{\tilde{y}\in Y_{x}}e^{S\left(X,\tilde{y}\right)}}, \end{array}$$

where *Y*_*x*_ is all possible label sequences of sentence *X*.

During training, we maximize the log likelihood of the correct label sequence:

(8) $$\begin{array}{c} \log\;P\left(y|X\right)=S\left(X,y\right)-\log\left(\sum_{\tilde{y}\in Y_{x}}e^{S\left(X,\tilde{y}\right)}\right). \end{array}$$

While decoding, we predict the sequence of labels with the highest conditional probability and use the Viterbi algorithm to decode the optimal label sequence.

(9) $$\begin{array}{c} y^{*}=\underset{\tilde{y}\in Y_{x}}{\text{argmax}}S\left(X,\tilde{y}\right). \end{array}$$

## 4. Results and Discussion

### 4.1. Dataset and Annotation Strategy

We run the experiments on the CCKS-2019 Task 1 benchmark dataset released by the 2019 China Conference on Knowledge Graph and Semantic Computing for a task about Chinese CNER. There are 1,000 records as the training dataset and 379 as the test dataset with six types of entities, i.e., disease, exam, test, operation, drug, and anatomy.  Table 1 lists the statistics of the entities of different types.

Here, we represent the entities with “BIO” (B-begin, I-inside, O-outside) tags in the following formats: B-X, I-X and O. B represents the starting position of the medical entity, I represents the remaining part of the medical entity, and O represents the nonmedical entity. *X* is the type of medical entity, which could be DIS, EXA, TES, OPE, DRU, and ANA.

### 4.2. Experimental Settings

Each clinical record may contain several sentences, leading to a too-long sample if we treat a record as a whole. Hence, we separate each record by a period to restrict the sentence length. After cutting the records, we set the maximum sequence length to 128. The IDCNN consists of 128 filters and the number of heads in MHA is 4. During training, we use the back-propagation algorithm and Adam optimizer with an initial learning rate of 3 × 10^−5^. The word embedding size is 128, and the activation function is ReLU. Also, the batch size is 20 and the dropout rate is 0.5.

### 4.3. Results and Analysis

#### 4.3.1. Comparison with Basic Models

To verify the effectiveness of the ALBERT-IDCNN-MHA-CRF, we compare the model with the following models:

- BiLSTM-CRF: a model based on BiLSTM and CRF. In this model, the dimension of the Word2Vec static word vector is 128.
- IDCNN-CRF: a model based on IDCNN and CRF. In this model, the dimension of the Word2Vec static word vector is 128.
- IDCNN–MHA-CRF: a model adding the MHA layer based on (b).
- ALBERT-IDCNN-CRF: a model adding the ALBERT pretraining model and fine-tuning based on (b).

Table 2 lists the experimental results of the different models. The experimental results show that our model's precision, recall and F1 score reach the highest values among the counterparts, with an increase of 3.67%, 3.15%, and 3.42%, respectively, from the baseline model, verifying the effectiveness of our model. The F1 scores of BiLSTM-CRF and IDCNN-CRF models are 81.27% and 81.49%, respectively, indicating that the recognition effects of the two models are equivalent. However, the 21-seconds-shorter per epoch running time demonstrates a better parallel computing power of IDCNN than BiLSTM. After adding the MHA layer, the F1 score increases by 0.99% (compared to 81.49%) and 1.33% (compared to 83.36%), respectively, which outlines the MHA's ability on extracting the contextual features. Also, replacing the traditional word vector model with fine-tuned ALBERT improves the F1 score by 1.87% (compared to 81.49%) and 2.21% (compared to 82.48%), respectively. This result has further strengthened our confidence that ALBERT has better semantic representation ability and has a more significant impact on the performance of the CNER task.

**Figure 3:**
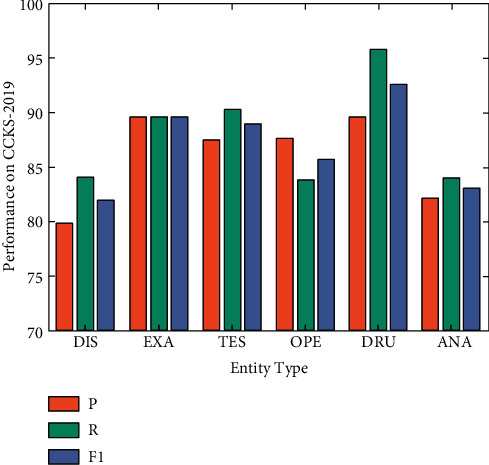
Results on different types of entities.

In addition to observing the evaluation metrics of the test dataset, we take a closer look at the predicted results.  Figure 3 reports the performance of the proposed model on different types of clinical entities. The plot reveals that the model performed well on drug and exam, reaching F1 scores of 92.62% and 89.66%, respectively, but fails to identify disease and anatomy effectively. After observing the errors, Table 3 lists the representative errors. First, these two types of entities are generally long, and supplementary information is in parentheses. For example, “(左肝)肝细胞性肝癌(中度分化)” ((left liver) hepatocellular carcinoma (moderately differentiated)), “腹主动脉旁淋巴结” (abdominal para-aortic lymph nodes). Therefore, when predicting this type of entity, there is a problem with boundary prediction errors, which leads to entity recognition errors. Second, some disease entities and operation entities are similar in text structure or nesting phenomena, resulting in the misclassification of this type of entity. As an illustration, among “胃癌” (gastric cancer) and “胃癌根治术” (radical gastrectomy for gastric cancer), the former belongs to the disease entity, while the latter is an operation entity. Third, the complex features of the two types of entities complicate the recognition.

#### 4.3.2. Comparison of Different Optimizers

We run the above experiment with Adam optimizer. Furthermore, we explore the influences of the Adagrad [25], RMSprop [26], Lookahead [27]+Adam, and RAdam [28] optimizers on entity recognition.  Table 4 presents how each optimizer improves the learning rate and gradient.

**Figure 4:**
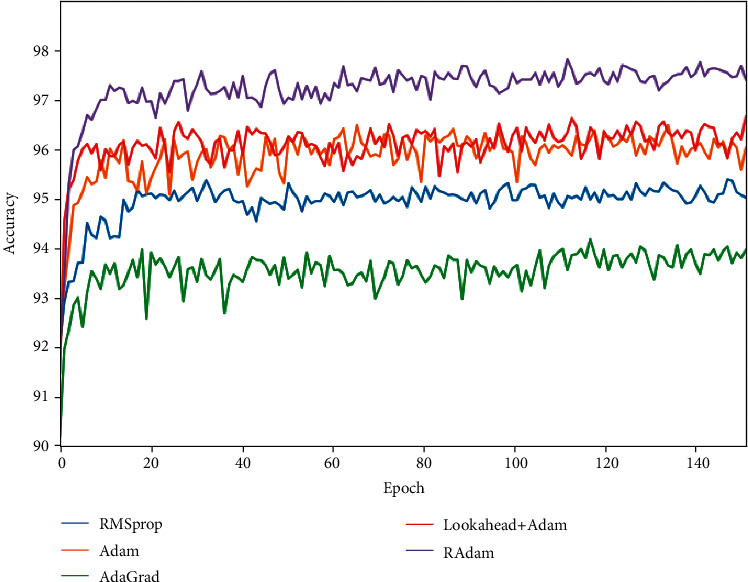
Comparison of the performance of different optimizers.

Applying the above optimizers to our model, Table 5 shows the experimental results, and Figure 4 shows the accuracy rate changes. The results identify that combining the dynamic adjustment of the gradient components is better than the one of dynamically adjusting the learning rate. Compared with the Adam baseline method, the performance is slightly improved after adding Lookahead, and its convergence speed is faster, which verifies the effectiveness of its exploration and integration strategy. We obtain the best model with the RAdam optimizer, whose F1 score reaches 85.63% and has an increase of 0.94% compared to Adam. The dynamic rectifier in RAdam adjusts Adam's adaptive momentum according to the variance and provides an automatic warm-up mechanism with regard to the dataset.

#### 4.3.3. Comparison with State-of-the-Art Models

Table 6 lists the test results of the other methods on the CCKS-2019 dataset [29]. The DUTIR team used the ELMO model to learn the contextual embedding representation of characters; then, it identified medical entities through the BiLSTM-CRF network; furthermore, it improved the model performance through transfer learning. The THU_MSIIP team used multiple different types of deep neural network models to complementarily introduce multiaspect information and used a postprocessing model based on dictionaries and context models to supplement. The Alihealth team proposed a method based on BERT and model fusion and constructed a series of rules through frequent pattern mining. However, the weak generality of those rules limited the scope of application. With RAdam optimizer, we achieve the best performance with an F1 score of 85.63%, and outperform other teams.

#### 4.3.4. Influence of the Number of Heads in MHA

**Figure 5:**
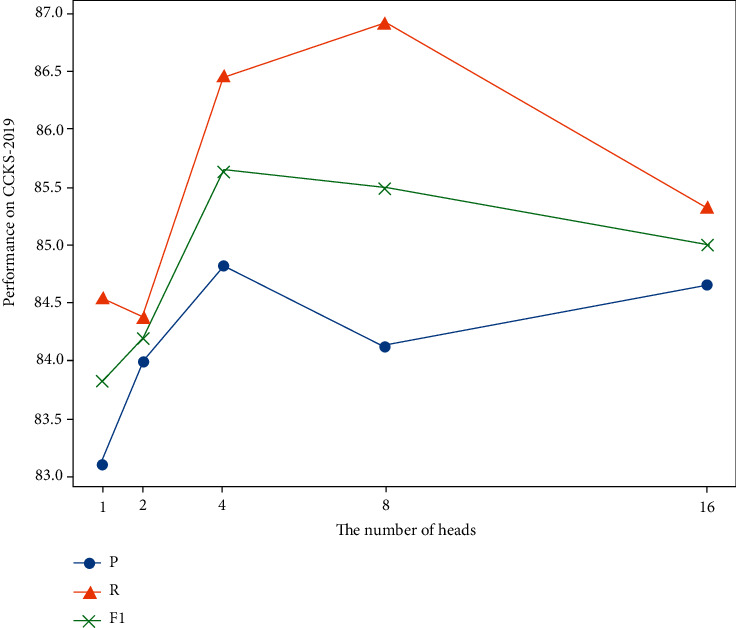
Influence of the number of heads.

The MHA layer can extract features from multiple aspects as different head can extract different features. To explore the influence of the most important hyperparameter on our model, recall that *h* is the number of heads, we set its value to 1, 2, 4, 8, and 16, respectively. We illustrate the results in Figure 5.

Figure 5 highlights the impact of *h*, where the performance improves as *h* increases from 1, since the text features are not fully extracted when *h* is small. On the other hand, the model learns too much redundant information when *h* is too large, harming the entity recognition. Therefore, by exploiting the value of *h*, we obtain the optimal performance when *h* is equal to 4.

## 5. Conclusions

This paper proposes a named entity recognition method, ALBERT-IDCNN-MHA-CRF, for the Chinese CNER task. The ALBERT pretraining language model more accurately represents contextual semantics in EMRs. Encoding entities through IDCNN achieves better recognition results, and the training speed has been improved. MHA captures rich semantic information in sentences. Furthermore, the RAdam optimizer benefits the performance. The proposed model achieves an F1 score of 85.63% on the CCKS-2019 dataset, superior to the state-of-the-art models. In future work, we will enrich the semantic representation of the embedding layer and introduce other features into the model. We will also consider the impact of nested entities to predict the boundaries of entities more accurately, thereby improving the overall entity recognition effect.

## Figures and Tables

**Table 1:** Statistics of different types of entities for the CCKS-2019.

	Disease	Exam	Test	Operation	Drug	Anatomy	Sum
Train	2116	222	318	765	456	1486	5363
Test	682	91	193	140	263	447	1816

**Table 2:** Results of different models.

Method	P (%)	R (%)	F1 (%)
BiLSTM-CRF(Baseline)	79.79	82.81	81.27
IDCNN-CRF	80.37	82.65	81.49
IDCNN-MHA-CRF	82.16	82.81	82.48
ALBERT-IDCNN-CRF	82.70	84.03	83.36
ALBERT-IDCNN-MHA-CRF	**83.46**	**85.96**	**84.69**

The best result on each metric is shown in bold face.

**Table 3:** Error samples.

Prediction	True entity
肝细胞性肝癌 (hepatocellular carcinoma)	(左肝)肝细胞性肝癌(中度分化) ((left liver) hepatocellular carcinoma (moderately differentiated))
胃癌 (gastric cancer)	胃癌根治术 (radical gastrectomy for gastric cancer)
肾上腺 (adrenal gland)	左肾上腺 (left adrenal gland)
淋巴结 (lymph nodes)	腹主动脉旁淋巴结 (abdominal para-aortic lymph nodes)

**Table 4:** Summary of different optimizers.

Optimizer	Year	Learning rate	Gradient
AdaGrad	2011	√	×
RMSprop	2012	√	×
Adam	2014	√	√
Lookahead + Adam	2019	√	√
RAdam	2019	√	√

“√” means dynamic adjustment, “×” means not.

**Table 5:** Results on different optimizers.

Optimizer	P (%)	R (%)	F1 (%)
AdaGrad	78.12	80.99	79.53
RMSprop	80.62	83.71	82.13
Adam	83.46	85.96	84.69
Lookahead + Adam	83.69	85.8	84.74
RAdam	**84.82**	**86.46**	**85.63**

The best result on each metric is shown in bold face.

**Table 6:** Comparison with state-of-the-art models.

Team name	Method	F1 (%)
Alihealth	BBC + BBT + FBBC + rule	85.62
THU_MSIIP	Ensemble	85.59
DUTIR	ELMO + BiLSTM-CRF	85.16
Jfhealthcare	—	84.85
Suda-hlt	—	84.12
ZJUCST	—	83.80
Ours	ALBERT-IDCNN-MHA-CRF	**85.63**

The best result is shown in bold face.

## Data Availability

The data used to support the findings of this study are available from the corresponding author upon request.
